# Effects of perinatal stress on the metabolites and lipids in plasma of dairy goats

**DOI:** 10.1007/s44154-023-00088-z

**Published:** 2023-05-12

**Authors:** Yan Huang, Yezi Kong, Bowen Li, Chenxu Zhao, Juan J. Loor, Panpan Tan, Yang Yuan, Fangyuan Zeng, Xiaoyan Zhu, Simeng Qi, Baoyu Zhao, Jianguo Wang

**Affiliations:** 1 College of Veterinary Medicine, Northwest A&F University, Yangling, 712100 Shaanxi China; 2 LipidALL Technologies Company Limited, Changzhou, 213022 Jiangsu China; 3 Department of Animal Sciences, Division of Nutritional Sciences, University of Illinois, Urbana, IL 61801 USA

**Keywords:** Dairy goat, Transition period, Untargeted metabolomics, Lipidomics

## Abstract

Dairy goats experience metabolic stress during the peripartal period, and their ability to navigate this stage of lactation is related to the occurrence and development of metabolic diseases. Unlike dairy cows, there is a lack of comprehensive analysis of changes in the plasma profiles of peripartal dairy goats, particularly using high-throughput techniques. A subset of 9 clinically-healthy dairy goats were used from a cohort of 96 primiparous Guanzhong dairy goats (BCS, 2.75 ± 0.15). Blood samples were collected at seven time points around parturition (d 21, 14, 7 before parturition, the day of kidding, and d 7, 14, 21 postpartum), were analyzed using untargeted metabolomics and targeted lipidomics. The orthogonal partial least squares discriminant analysis model revealed a total of 31 differential metabolites including p-cresol sulfate, pyruvic acid, cholic acid, and oxoglutaric acid. The pathway enrichment analysis identified phenylalanine metabolism, aminoacyl-tRNA biosynthesis, and citrate cycle as the top three significantly-altered pathways. The Limma package identified a total of 123 differentially expressed lipids. Phosphatidylserine (PS), free fatty acids (FFA), and acylcarnitines (ACs) were significantly increased on the day of kidding, while diacylglycerols (DAG) and triacylglycerols (TAG) decreased. Ceramides (Cer) and lyso-phosphatidylinositols (LPI) were significantly increased during postpartum period, while PS, FFA, and ACs decreased postpartum and gradually returned to antepartum levels. Individual species of FFA and phosphatidylcholines (PC) were segregated based on the differences in the saturation and length of the carbon chain. Overall, this work generated the largest repository of the plasma lipidome and metabolome in dairy goats across the peripartal period, which contributed to our understanding of the multifaceted adaptations of transition dairy goats.

**Supplementary Information:**

The online version contains supplementary material available at 10.1007/s44154-023-00088-z.

## Introduction

Milk production in the dairy goat industry has more than doubled in the last 50 years (Pulina et al. 2018). The metabolic status of dairy goats in the perinatal period is extremely important for milk production and quality (Stelletta et al. 2008; Matthews 2016). Similar to cows, dairy goats experience dramatic changes in energy demands during the transition into lactation and are highly-susceptible to negative energy balance (NEB) (Bell 1995; Simões and Gutiérrez 2017). The metabolic pressure can trigger common metabolic diseases such as hypocalcemia (milk fever), fatty liver syndrome, and ketosis in dairy cows (Adewuyi et al. 2005; McCarthy et al. 2015; Ringseis et al. 2015). Our previous study also detected a sustained state of oxidative stress in dairy goats during the around parturition (Huang et al. 2021). Metabolic diseases that occur in the peripartal period might affect milk production, quality, and animal welfare, all of which delay the resumption of estrous cyclicity, and even milk production in the subsequent lactation (McArt et al. 2012; Ribeiro et al. 2013; Sordillo and Raphael 2013; Raboisson et al. 2014). Thus, peripartal health management programs for dairy goats are critical for animal welfare and economic outcomes.

Although excessive lipolysis is presently recognized as an important factor for developing ketosis and fatty liver, the molecular basis of successful or impaired adaptations to the metabolic challenges in early lactation dairy goats remains incomplete (Ceciliani et al. 2018). In recent years, metabolomics has become a useful tool for understanding the disease pathophysiology and contributing to identify disease biomarkers for use in preventive protocols (Saleem et al. 2012; Hailemariam et al. 2014; Ceciliani et al. 2018; Wang et al. 2020). Untargeted metabolomics involves the qualitative determination of chemical signatures in a biological sample such as blood (Schrimpe-Rutledge et al. 2016). In contrast, targeted metabolomics analyzes specific metabolite clusters associated with certain metabolic pathways such as lipid species. Using untargeted metabolomics, a recent study identified variations in the metabolome, indicated enrichment in pathways such as lipid, glucose (GLU), and nucleotide metabolism after calving along with a decrease in amino acid metabolism (Luo et al. 2019). Some studies have also identified changes in acylcarnitines (ACs), glycerophospholipid, and sphingomyelin in the blood of peripartal dairy cows (Kenez et al. 2016). However, very little is known about metabolome profiles in peripartal dairy goats.

The present study used untargeted metabolomics and targeted lipidomics for analyzing plasma metabolite profiles in peripartal dairy goats at seven crucial time points (d 21, 14, 7 before parturition, on the kidding day, and d 7, 14, 21 postpartum). The objectives of this investigation involved (1) clarifying the metabolic changes in small molecule metabolites during the peripartal period; and (2) identifying potential biomarkers for characterizing new pathways that might be perturbed under metabolic stress.

## Results

### Plasma biochemical indices

**Fig. 1:**
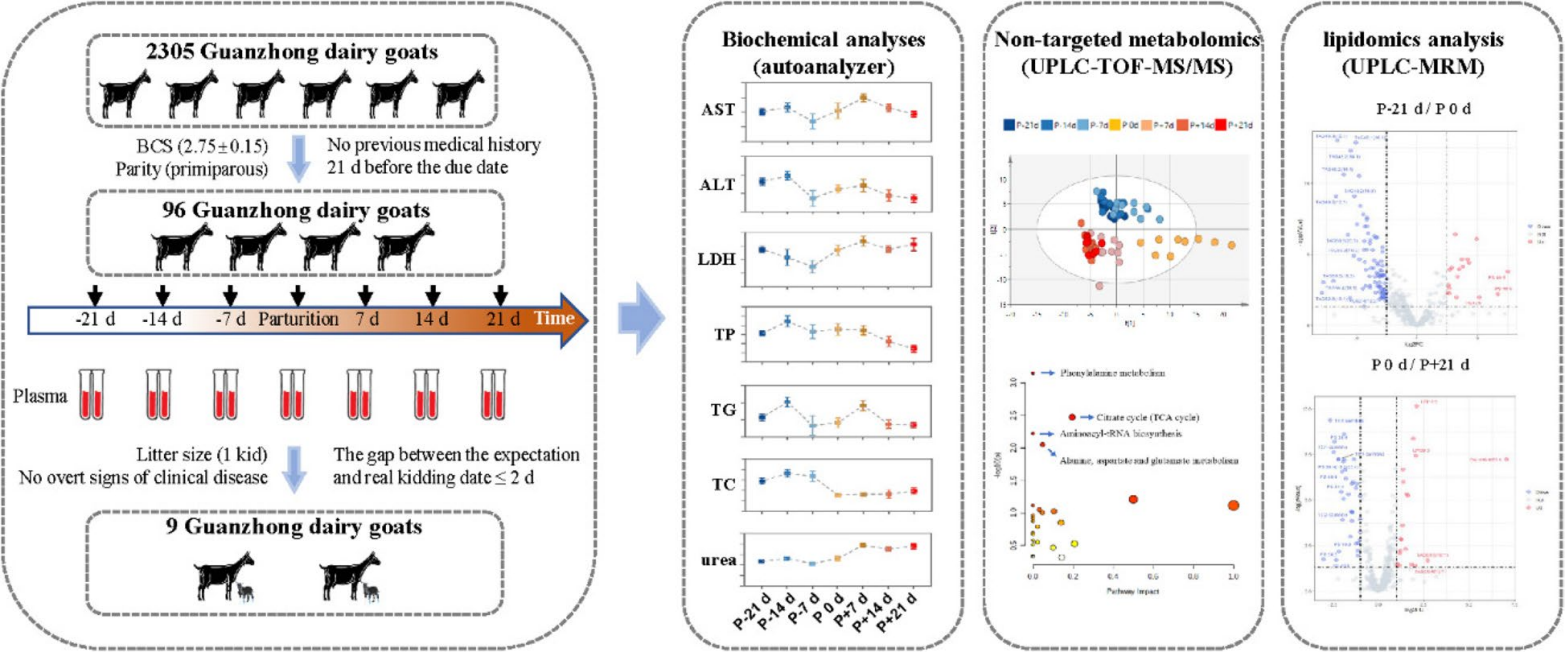
Overview of the experimental design. Ninety-six multiparous healthy Guanzhong dairy goats (primiparous, Shaanxi, China) of similar age, BCS, and due date were selected. The subset of goats used was selected to include only healthy animals with a gap between expected and real kidding day of two days or less. Subsequently, A subset of 9 clinically-healthy dairy goats were used

An overview of the experimental design is shown in Fig. 1. Table 1 presents a summary of the biochemical data. Observations indicate significant changes in the plasma biochemical indices (non–esterified fatty acids [NEFA], β-Hydroxybutyrate [BHB], aminotransferase [AST], alanine aminotransferase [ALT], lactate dehydrogenase [LDH], total protein [TP], triglyceride [TG], total cholesterol [TC], urea, *P* < 0.05) during the peripartal period. Antepartum, plasma NEFA levels showed an increase, followed by a significant decrease postpartum. Plasma BHB levels displayed a significant increase at P-14 d and displayed an upward trend throughout the perinatal period. The goats exhibited low levels of plasma AST and LDH at P-7 d, subsequently reaching maximum levels at P + 7 d. Plasma ALT and TG levels presented a significant decrease at P-7 d with a sustained low level thereafter. Plasma TP levels indicated a decrease at P + 14 d and reached minimum levels at P + 21 d. Plasma TC levels showed a significant decrease at P 0 d and remained low over the observation period. Meanwhile, the plasma urea levels displayed a statistically significant increase at P + 7 d (*P* < 0.05) and persisted at high levels until the observation period's end.

**Table 1:** Biochemical data of dairy goats during the perinatal period^1^

	Day relative to kidding						
Biomarker^2^	P-21 d	P-14 d	P-7 d	P 0 d	P + 7 d	P + 14 d	P + 21 d
NEFA* (mmol/L)	0.273 ± 0.068^b^	0.200 ± 0.046^b^	0.438 ± 0.048^ab^	0.691 ± 0.182^a^	0.162 ± 0.066^b^	0.183 ± 0.028^b^	0.146 ± 0.031^b^
BHB* (mmol/L)	0.253 ± 0.017^c^	0.323 ± 0.012 ^bc^	0.305 ± 0.022 ^bc^	0.401 ± 0.032^b^	0.455 ± 0.044^ab^	0.520 ± 0.041^ab^	0.544 ± 0.037^a^
GLU (μmol/L)	3.22 ± 0.087	3.76 ± 0.186	3.3 ± 0.392	4.04 ± 0.586	4.16 ± 0.301	4.18 ± 0.190	3.76 ± 0.129
AST* (U/L)	80.6 ± 4.28^ab^	86.2 ± 5.97^ab^	67.4 ± 10.0^b^	81.8 ± 10.9^ab^	99.8 ± 5.20^a^	85.6 ± 5.00^ab^	77.2 ± 4.19^ab^
ALT* (U/L)	18.2 ± 1.01^ab^	19.8 ± 1.21^a^	13.6 ± 2.19^b^	16.2 ± 1.02^ab^	17.2 ± 1.67^ab^	14.4 ± 1.51^ab^	13.6 ± 1.07^b^
AST/ALT	4.51 ± 0.26	4.40 ± 0.26	4.63 ± 0.62	5.28 ± 0.67	6.18 ± 0.53	6.57 ± 0.77	5.90 ± 0.41
CHE (U/L)	124 ± 5.83	134 ± 7.31	107 ± 13.9	114 ± 9.20	141 ± 10.3	129 ± 7.85	119 ± 7.07
GGT (U/L)	54.8 ± 2.93	59.4 ± 3.58	46.0 ± 7.44	52.2 ± 3.63	61.0 ± 3.75	65.8 ± 4.93	62.8 ± 4.553
LDH* (U/L)	289 ± 11.2^ab^	259 ± 29.1^ab^	226 ± 23.5^b^	287 ± 19.2^ab^	319 ± 18.2^a^	290 ± 12.1^ab^	307 ± 22.9^ab^
ALP (U/L)	263 ± 39.2	292 ± 59.5	149 ± 30.6	159 ± 26.9	127 ± 28.3	150 ± 27.6	162 ± 39.0
TP* (g/L)	45.6 ± 1.36^ab^	52.2 ± 3.02^a^	46.6 ± 3.69^ab^	47.8 ± 3.27^ab^	47.4 ± 2.55^ab^	41.2 ± 2.74^ab^	37.2 ± 1.77^b^
ALB (g/L)	26.5 ± 0.786	29.5 ± 1.37	26.8 ± 2.74	27.1 ± 1.61	29.7 ± 1.34	27.5 ± 1.47	25.5 ± 0.758
GLB (g/L)	67.2 ± 1.47	74.9 ± 3.34	66.7 ± 6.82	68.7 ± 3.68	81.7 ± 4.05	75.6 ± 3.07	69.1 ± 2.05
HDL (mmol/L)	1.17 ± 0.065	1.48 ± 0.087	1.32 ± 0.157	1.25 ± 0.089	1.38 ± 0.086	1.23 ± 0.077	1.13 ± 0.051
LDL (μmol/L)	628 ± 44.2	742 ± 71.3	642 ± 71.5	540 ± 50.1	734 ± 73.9	518 ± 41.4	542 ± 39.7
TG* (mmol/L)	2.06 ± 0.090^b^	2.52 ± 0.141^a^	1.84 ± 0.281^b^	1.92 ± 0.141^ab^	2.41 ± 0.151^ab^	1.88 ± 0.126^ab^	1.85 ± 0.083^ab^
TC* (μmol/L)	288 ± 30.0^abc^	362 ± 38.2^a^	334 ± 49.3^ab^	154 ± 18.4^c^	162 ± 14.4^c^	164 ± 40.3^c^	196 ± 29.3^bc^
urea* (μmol/L)	4.66 ± 0.233^b^	5.29 ± 0.361^b^	4.23 ± 0.315^b^	5.25 ± 0.530^b^	7.78 ± 0.399^a^	7.08 ± 0.390^a^	7.62 ± 0.507^a^

^1^Data are expressed as mean ± SE (*n* = 9/group). Mean values with different letters (a–c) in rows show statistically significant differences (*P* < 0.05). *ANOVA *P*-value < 0.05. P-21 d, P-14 d, P-7 d (d 21,14 and 7 before the due date), P 0 d (the day of kidding), and P + 7 d, P + 14 d, P + 21 d (d 7, 14, and 21postpartum)

^2^*GLU* Glucose, *AST* Aspartate aminotransferase, *ALT* Alanine aminotransferase, *CHE* Cholinesterase, *GGT* γ-glutamyl transpeptidase, *LDH* Lactate dehydrogenase, *ALP* Alkaline phosphatase, *TP* Total protein, *ALB* Albumin, *GLB* Globulin, *HDL* High density cholesterol, *LDL* Low density cholesterol, *TG* Triglyceride, *TC* Total cholesterol

Table 1 summarizes the biochemical data. The biochemical indices (NEFA, BHB, AST, ALT, LDH, TP, TG, TC, urea, *P* < 0.05) in plasma changed significantly during the peripartal period. The plasma NEFA levels was increased antepartum and then rapidly decreased postpartum. The plasma BHB levels increased significantly at P-14 d and maintained an upward trend over the perinatal period. The goats exhibited low plasma AST and LDH levels at P-7 d, and reached maximum levels at P + 7 d. The plasma ALT and TG levels decreased significantly at P-7 d and remained low thereafter. The plasma TP levels decreased at P + 14 d and reached the minimum levels at P + 21 d. The plasma TC levels were found to decrease significantly at P 0 d and remained low thereafter. The plasma urea levels increased significantly at P + 7 d and remained high until the end of the experiment (Fig. 1).

### Untargeted metabolomics analysis

**Fig. 2:**
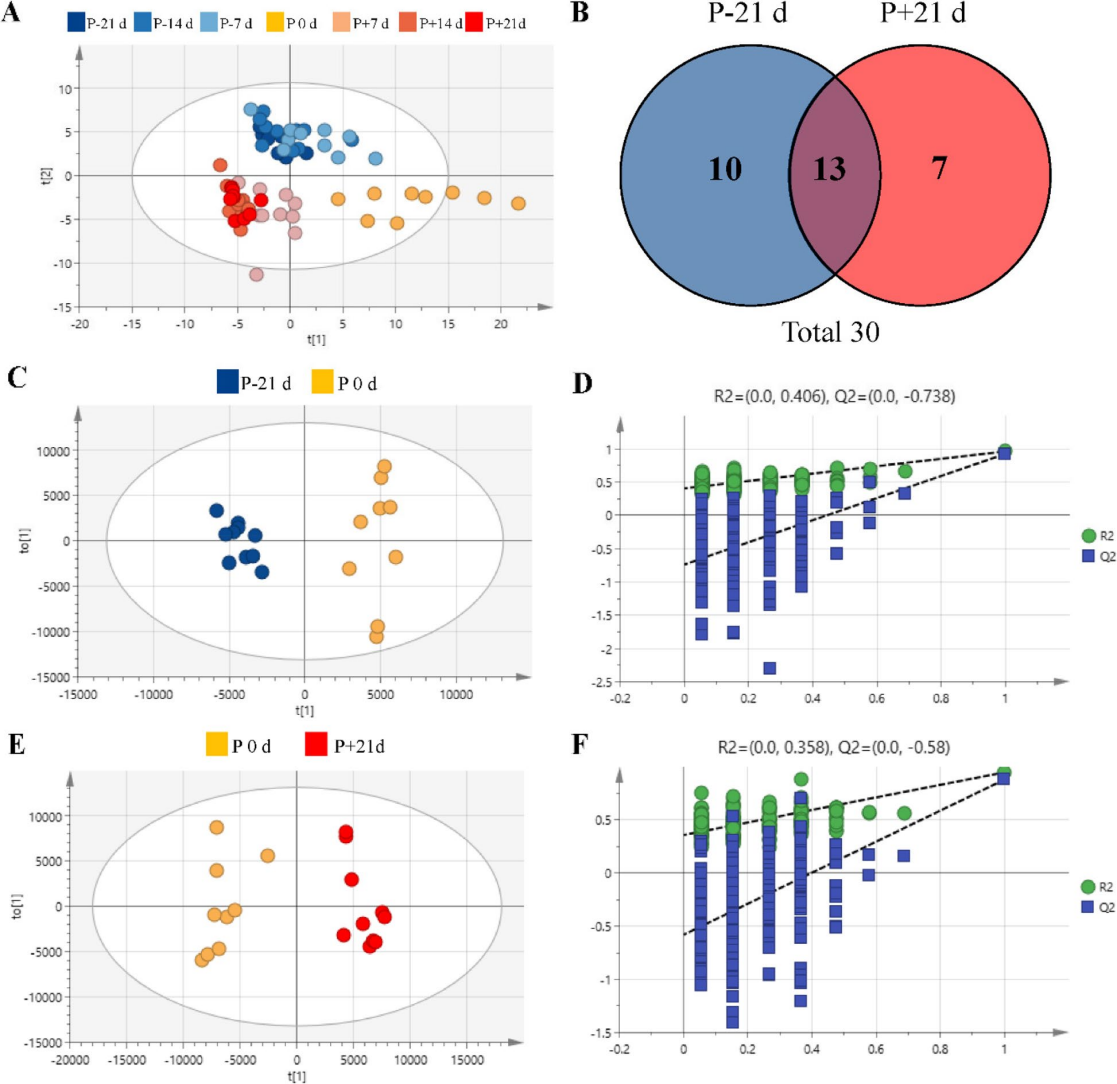
Metabolite profiles of perinpartal dairy goats: **A** principal component analysis (PCA) score plot for the seven groups, **B** Venn diagram analyses for differential metabolites, **C** and **D** orthogonal partial least squares discriminant analysis (OPLS-DA) score plot and permutation test plots for P-21 d vs. P 0 d, **E** and **F** OPLS-DA score plot and permutation test plots for P 0 d vs. P + 21d. t[1], first principal component. to[2], second orthogonal component. The intercept limit of Q^2^, calculated by the regression line, is the plot of Q.^2^ from the permutation test in the OPLS-DA model. P-21 d, P-14 d, P-7 d (d 21,14 and 7 before the due date), P 0 d (the day of kidding), and P + 7 d, P + 14 d, P + 21 d (d 7, 14, and 21postpartum)

A total of 156 different metabolites were identified and quantified after quality control (QC). The principal component analysis (PCA) score plot (Fig. 2A) shows the distribution of samples from the 7-time points. Three groups before kidding and the other three groups after kidding were clustered together separately with no intersection and were separated from the P 0 d group. The orthogonal partial least squares discriminant analysis (OPLS-DA) analysis and the permutation tests (Fig. 2C and E) showed that based on the OPLS-DA models, the P-21 d and P 0 d groups were discriminated with R^2^Y = 0.963 and Q^2^ = 0.925. On the other hand, the P + 21d and P 0 d groups were discriminated with R^2^Y = 0.948 and Q^2^ = 0.88. There was a clear separation with no overlap for the OPLS-DA plots of the plasma metabolomics data. The stability and reliability of the OPLS-DA model were confirmed by the satisfactory explanatory and predictive values for the intercepts (R^2^, Q^2^) of the permutation testing (Fig. 2D and F).

**Fig. 3:**
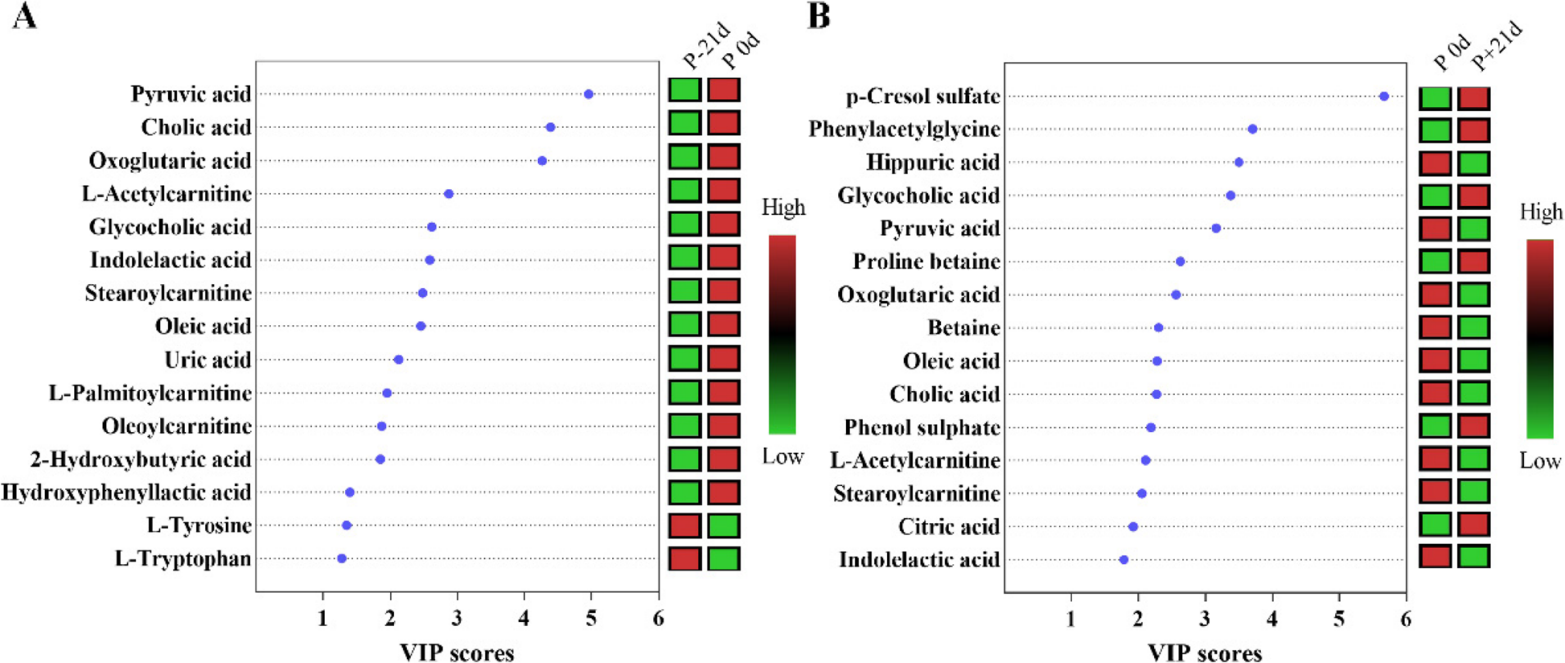
Rank order of the top 15 discriminating metabolites by variable importance in projection (VIP) scores: **A** P-21 d vs. P 0 d, **B** P 0 d vs. P + 21d. P-21 d, d 21 before the due date; P 0 d, the day of kidding; P + 21 d, d 21postpartum

The high variable importance in projection (VIP) scores indicated the metabolites contributed greatly to the group separation. Based on the VIP value in the OPLS-DA model (VIP > 1) and *P*-value in the student’s t-test (*P* < 0.05), 23 and 20 differential metabolites were identified in P 0 d vs. P-21 d and P 0 d vs. P + 21 d groups, respectively (Table 2 and Fig. 2B). The VIP score plot highlighted the 15 top-scoring metabolites (Fig. 3). Most metabolites were more abundant in the P 0 d group, such as pyruvic acid, cholic acid, oxoglutaric acid, L-acctylcarnitine, indolelactic acid, stearoylcarnitine, oleic acid, compared to P + 21 d and P-21 d group, respectively. There were also changes in the differential metabolites during the peripartal period as demonstrated in Fig. S2 as box plots.

**Table 2:** List of VIP scores of OPLS-DA and *P*-value for the non-targeted metabolomics models^a^

P-21 d vs. P 0 d			P + 21 d vs. P 0 d		
Metabolites	VIP	*P* value	Metabolites	VIP	*P* value
Pyruvic acid	4.956	0.0196	p-Cresol sulfate	5.667	0.0165
Cholic acid	4.388	0.0064	Phenylacetylglycine	3.709	0.0003
Oxoglutaric acid	4.266	< 0.0001	Hippuric acid	3.499	0.0140
L-Acetylcarnitine	2.868	0.0003	Glycocholic acid	3.376	0.0478
Glycocholic acid	2.615	0.0110	Pyruvic acid	3.159	0.0373
Indolelactic acid	2.586	< 0.0001	Proline betaine	2.627	< 0.0001
Stearoylcarnitine	2.480	< 0.0001	Oxoglutaric acid	2.556	< 0.0001
Oleic acid	2.453	0.0015	Betaine	2.302	< 0.0001
Uric acid	2.122	< 0.0001	Oleic acid	2.276	< 0.0001
L-Palmitoylcarnitine	1.947	< 0.0001	Cholic acid	2.269	0.0171
Oleoylcarnitine	1.867	< 0.0001	Phenol sulphate	2.188	0.0006
2-Hydroxybutyric acid	1.849	0.0003	L-Acetylcarnitine	2.106	0.0002
Hydroxyphenyllactic acid	1.395	< 0.0001	Stearoylcarnitine	2.055	< 0.0001
L-Tyrosine	1.346	0.0042	Citric acid	1.925	0.0265
L-Tryptophan	1.276	< 0.0001	Indolelactic acid	1.783	0.0001
2-Ethylhydracrylic acid	1.275	0.0001	L-Palmitoylcarnitine	1.593	< 0.0001
L-Carnitine	1.270	0.0002	Oleoylcarnitine	1.542	< 0.0001
C17-carnitine	1.257	0.0006	Uric acid	1.406	< 0.0001
L-Valine	1.240	0.0012	Indoxyl sulfate	1.388	0.0428
Proline betaine	1.148	0.0001	Linoleic acid	1.075	< 0.0001
L-Methionine	1.139	0.0128			
L-Threonic Acid	1.135	0.0015			
Linoleic acid	1.135	0.0005			

^a^*VIP* Variable importance in projection, *OPLS-DA* Orthogonal partial least squares discriminant analysis, *P* values refer to paired t‐test. P-21 d (d 21 before the due date), P 0 d (the day of kidding), and P + 21 d (d 21postpartum)

### Functional annotation and pathway enrichment

**Fig. 4:**
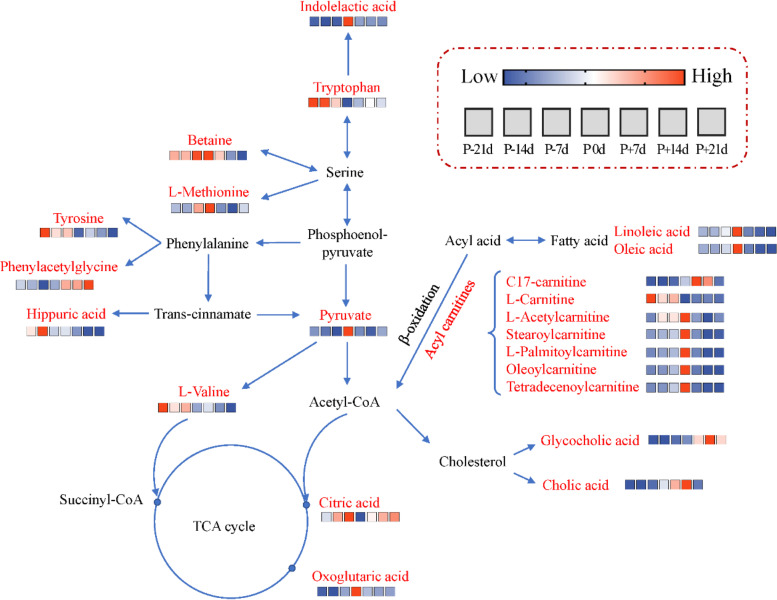
Differentially altered metabolites in lipid metabolism, energy metabolism, and amino metabolism pathways. The colors of the cells indicate the relative change during the peripartal period. P-21 d, P-14 d, P-7 d (d 21,14 and 7 before the due date), P 0 d (the day of kidding), and P + 7 d, P + 14 d, P + 21 d (d 7, 14, and 21postpartum)

**Fig. 5:**
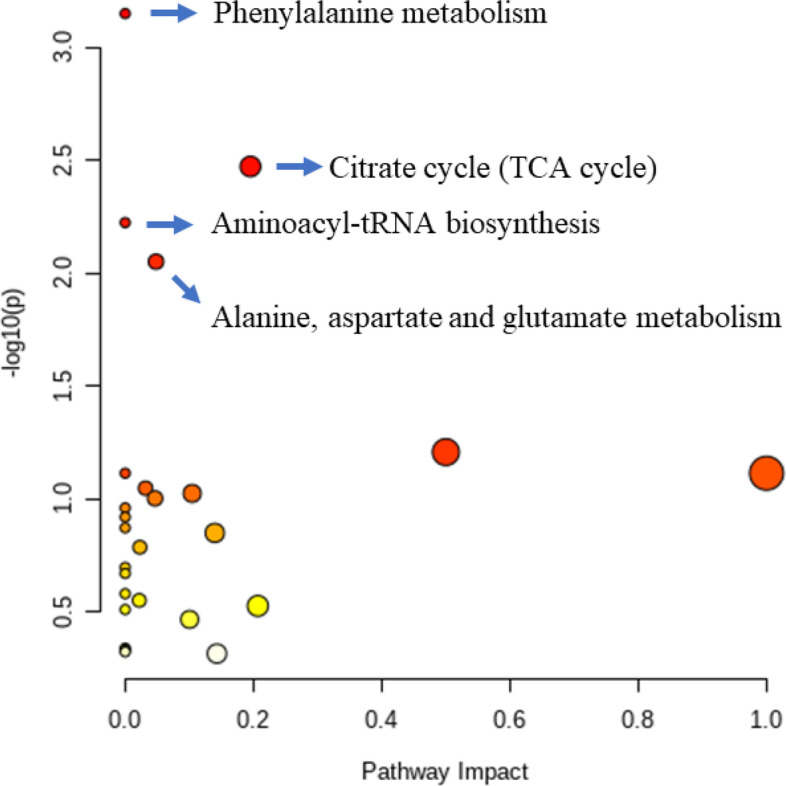
Metabolic pathway analysis using MetaboAnalyst 4.0. Circles represent metabolic pathways. Darker circles indicate more significant changes for metabolites in the corresponding pathway, whereas the size of the circle corresponds to the pathway impact score

Differentially altered metabolites were analyzed through the KEGG Metabolome Database and MetaboAnalyst. They were marked based on published articles and KEGG analysis (Fig. 4). The relative variation of differentially altered metabolites during the peripartal period was displayed in the form of heat maps in the metabolic network. To gain further insight into the changes in metabolic processes during the peripartal period, the pathway enrichment was analyzed using significantly-altered metabolites (Fig. 5). Here, phenylalanine metabolism, aminoacyl-tRNA biosynthesis, and citrate cycle (TCA cycle) were identified as significant pathways.

### Targeted lipidomics analysis

**Fig. 6:**
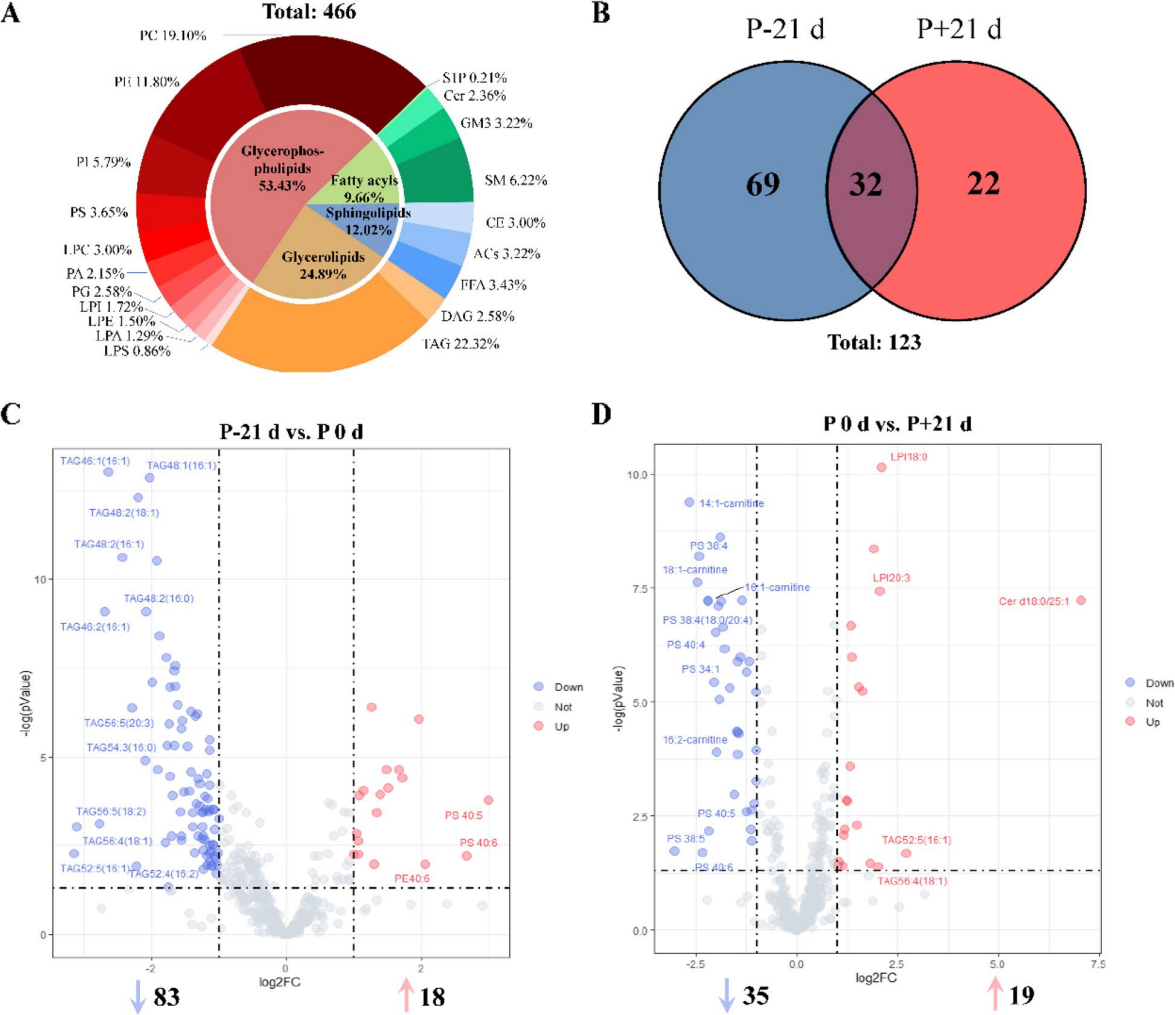
Plasma target lipidomic analysis of transition dairy goats: **A** Percentage of lipid species, **B** Venn diagram analyses of differentially altered lipids, **C** Volcano plot showing differentially altered lipids between P-21 d (d 21 before the due date) and. P 0 d (the day of kidding), **D** Volcano plot showing differentially altered lipids between P 0 d and P + 21d (d 21postpartum). Differential altered lipids (|log2FC|> 2) are highlighted with lipid names. The two dashed vertical lines indicate log2FC of -1 (left) and 1 (right). The dashed horizontal line indicates a false discovery rate (FDR) of 0.05. ACs, acylcarnitine; CE, cholesteryl esters; Cer, ceramides; DAG, diacylglycerols; FFA, free fatty acids; GM3, monosialogangliosides; PA, phosphatidic acids; PC, phosphatidylcholines; LPC, lyso-PC; PE, phosphatidylethanolamines; PG, phosphatidylglycerols; PI, phosphatidylinositols; PS, phosphatidylserines; S1P, sphingosine-1-phosphate; SM, sphingomyelins; TAG, triacylglycerols

Numerous fatty acids and ACs related to beta-oxidation and mobilization of fat storage were identified as statistically significant. Thus, high-coverage lipidomics was performed for elucidating changes in lipid metabolism during parturition in dairy goats. The lipidomic analysis quantitated 466 lipids spanning 20 lipid classes (Fig. 6A). The TAG (*n* = 104) followed by PC (*n* = 89), PE (phosphatidylethanolamines, *n* = 55) and SM (Sphingomyelins, *n* = 29) had the highest diversity of individual lipid molecular species. Changes in each lipid subclass during the peripartal period are shown in Fig. S3. The differentially expressed lipids were screened by determining |log2FC|> 1 and FDR < 0.05 from Limma model (Fig. 6C and D). There were 101 and 54 differentially expressed lipids in P 0 d vs. P-21 d and P 0 d vs. P + 21 d groups, respectively (Table 3 and Fig. 6B). Compared to P-21 d, 12 TAG were significantly reduced (log2FC < -2) at P 0 d, while PS 40:5, PS 40:6 and PE 40:6 were significantly increased (log2FC > 2). Compared to P 0 d, there was significant reduction in 4 ACs and 7 PS (log2FC < -2) at P + 21 d, while LPI 18:0, LPI 20:3, Cer d(18:0/25:1), TAG 52:5(16:1), and TAG 56:4(18:1) were significantly increased (log2FC > 2) (Table 4).

**Table 3:** List of differentially altered lipids in P-21 d vs. P 0 d (|log2FC|> 1, FDR < 0.05)^a^

Lipid^b^	Log2FC	FDR	Lipid	Log2FC	FDR
12:0-carnitine	1.04	0.0014	TAG50:1(18:1)	-1.14	0.0003
14:0-carnitine	1.14	0.0001	TAG50:2(16:1)	-1.78	< 0.0001
14:1-carnitine	1.51	0.0001	TAG50:2(18:1)	-1.55	< 0.0001
16:0-carnitine	1.26	< 0.0001	TAG50:3(16:0)	-1.21	0.0001
16:1-carnitine	1.66	< 0.0001	TAG50:3(16:1)	-1.66	< 0.0001
16:2-carnitine	1.30	0.0106	TAG50:3(18:1)	-1.15	< 0.0001
17:0-carnitine	1.39	0.0001	TAG50:3(18:2)	-1.21	0.0003
18:0-carnitine	1.48	< 0.0001	TAG50:4(16:2)	-1.76	0.0461
18:1-carnitine	1.72	< 0.0001	TAG51:0(17:0)	-1.32	< 0.0001
FFA18:1	1.06	0.0024	TAG51:2(15:0)	-1.76	< 0.0001
FFA18:2	1.08	0.0001	TAG51:2(17:0)	-1.33	0.0002
FFA22:6	1.00	0.0057	TAG51:2(17:1)	-1.75	< 0.0001
PE40:6	2.06	0.0106	TAG51:3(17:1)	-1.73	< 0.0001
PS 38:4(18:0/20:4)	1.07	0.0057	TAG52:0(18:0)	-1.32	0.0017
PS 40:4	1.34	0.0004	TAG52:1(16:1)	-1.52	0.0001
PS 40:5	3.00	0.0002	TAG52:1(18:1)	-1.24	0.0020
PS 40:5(18:0/22:5)	1.96	< 0.0001	TAG52:2(16:0)	-1.09	0.0003
PS 40:6	2.67	0.0061	TAG52:2(16:1)	-1.24	0.0004
PG38:3(18:0/20:3)	-1.11	0.0117	TAG52:2(18:1)	-1.06	0.0011
LPI16:0	-1.14	< 0.0001	TAG52:2(18:2)	-1.02	0.0111
LPI16:1	-1.41	< 0.0001	TAG52:3(16:1)	-1.47	< 0.0001
LPI18:0	-1.65	< 0.0001	TAG52:4(16:1)	-1.44	0.0001
LPI20:3	-1.99	< 0.0001	TAG52:4(16:2)	-2.23	0.0120
LPI20:4	-1.65	< 0.0001	TAG52:5(16:1)	-3.15	0.0053
LPI22:4	-1.41	< 0.0001	TAG52:5(18:2)	-1.04	0.0188
LPS18:0	-1.28	0.0002	TAG53:2(17:0)	-1.28	0.0001
LPS18:1	-1.14	0.0031	TAG53:2(19:1)	-1.13	0.0044
LPC20:3	-1.00	0.0005	TAG53:3(17:1)	-1.09	0.0031
LPC20:5	-1.24	0.0009	TAG53:4(17:0)	-1.36	0.0050
LPC22:3	-1.57	0.0004	TAG53:4(17:1)	-1.01	0.0049
PA32:1	-1.25	0.0004	TAG54:0(18:0)	-1.56	0.0017
PA32:2	-1.23	0.0150	TAG54:1(18:0)	-1.70	0.0017
PC40:6(20:3/20:3)	-1.21	0.0022	TAG54:1(18:1)	-1.55	0.0022
DAG32:1(14:0/18:1)	-1.14	0.0001	TAG54:2(18:0)	-1.14	0.0091
DAG32:1(16:1/16:0)	-1.61	< 0.0001	TAG54:2(18:1)	-1.18	0.0062
DAG36:1(18:1/18:0)	-1.18	< 0.0001	TAG54:2(18:2)	-1.11	0.0111
DAG36:2(18:1/18:1)	-1.35	< 0.0001	TAG54:3(16:0)	-2.09	< 0.0001
TAG46:1(16:0)	-1.88	< 0.0001	TAG54:4(16:0)	-1.91	< 0.0001
TAG46:1(16:1)	-2.63	< 0.0001	TAG54:4(18:3)	-1.23	0.0041
TAG46:2(16:1)	-2.69	< 0.0001	TAG54:5(16:0)	-1.05	0.0072
TAG48:1(16:0)	-1.92	< 0.0001	TAG54:5(20:4)	-1.15	0.0004
TAG48:1(16:1)	-2.03	< 0.0001	TAG56:3(18:1)	-1.15	0.0053
TAG48:1(18:0)	-1.69	0.0001	TAG56:3(20:1)	-1.79	0.0026
TAG48:1(18:1)	-1.55	< 0.0001	TAG56:4(18:1)	-3.11	0.0009
TAG48:2(16:0)	-2.07	< 0.0001	TAG56:5(18:1)	-1.73	< 0.0001
TAG48:2(16:1)	-2.43	< 0.0001	TAG56:5(18:2)	-2.78	0.0008
TAG48:2(18:1)	-2.20	< 0.0001	TAG56:5(20:3)	-2.28	< 0.0001
TAG48:2(18:2)	-1.24	0.0017	TAG56:6(20:3)	-1.39	0.0009
TAG50:1(16:0)	-1.17	0.0002	TAG56:6(20:4)	-1.07	0.0062
TAG50:1(16:1)	-1.66	< 0.0001	TAG56:7(20:5)	-1.18	0.0113
TAG50:1(18:0)	-1.39	0.0004			

^a^Fold changes (FC) were calculated as the average levels in the P 0 d (the day of kidding) group relative to those in the P-21 d (d 21 before the due date) group. The Log2FC greater than 0 indicates a relatively higher concentration in the P 0 d group, whereas the Log2FC of less than 0 indicates a concentration lower than that in the P-21 d group. *FDR* False discovery rate

^b^*FFA* Free fatty acids, *PE* Phosphatidylethanolamines, *PS* Phosphatidylserines, *PG* Phosphatidylglycerols, *LPI* Lyso-phosphatidylinositols, *PC* Phosphatidylcholines, *LPC* Lyso- phosphatidylcholines, *PA* Phosphatidic acids, *DAG* Diacylglycerols, *TAG* Triacylglycerols

**Table 4:** List of differentially altered lipids in P 0 d vs. P + 21 d (|log2FC|> 1, FDR < 0.05)^a^

Lipid^b^	Log2FC	FDR	Lipid	Log2FC	FDR
Cer d18:0/25:1	7.03	< 0.0001	PS 38:4	-2.43	< 0.0001
LPI16:0	1.34	< 0.0001	PS 38:4(18:0/20:4)	-2.21	< 0.0001
LPI16:1	1.54	< 0.0001	PS 38:5	-3.04	0.0185
LPI18:0	2.10	< 0.0001	PS 40:4	-2.01	< 0.0001
LPI20:3	2.06	< 0.0001	PS 40:5	-2.19	0.0066
LPI20:4	1.89	< 0.0001	PS 40:5(18:0/22:5)	-1.48	0.0001
LPI22:4	1.36	< 0.0001	PS 40:6	-2.35	0.0202
LPC20:5	1.22	0.0014	12:0-carnitine	-1.88	< 0.0001
PA32:1	1.63	< 0.0001	14:0-carnitine	-1.92	< 0.0001
PA32:2	1.11	0.0387	14:1-carnitine	-2.66	< 0.0001
PC42:6(22:6/20:3)	1.32	0.0002	14:2-carnitine	-1.46	< 0.0001
TAG52:5(16:1)	2.70	0.0206	16:0-carnitine	-1.36	< 0.0001
TAG54:3(16:0)	1.01	0.0385	16:1-carnitine	-2.21	< 0.0001
TAG56:4(18:1)	2.02	0.0399	16:2-carnitine	-2.01	0.0001
TAG56:4(18:2)	1.04	0.0311	17:0-carnitine	-1.66	< 0.0001
TAG56:5(18:2)	1.82	0.0349	17:1-carnitine	-1.92	< 0.0001
TAG56:5(20:3)	1.17	0.0081	18:0-carnitine	-1.84	< 0.0001
TAG56:6(20:3)	1.17	0.0062	18:1-carnitine	-2.48	< 0.0001
SM d18:1/15:1	1.24	0.0015	18:2-carnitine	-1.49	< 0.0001
SM d18:1/26:1	-1.25	0.0025	6:0-carnitine	-1.48	< 0.0001
PE36:2p	-1.14	0.0062	FFA17:0	-1.02	< 0.0001
PE38:4p	-1.12	0.0110	FFA18:1	-1.80	< 0.0001
PE38:4p(18:0/20:4)	-1.01	0.0005	FFA18:2	-1.40	< 0.0001
PS 34:1	-2.07	< 0.0001	FFA18:3	-1.26	< 0.0001
PS 36:2	-1.47	< 0.0001	FFA20:4	-1.00	0.0001
PS 38:3	-1.96	< 0.0001	FFA22:5	-1.07	0.0016
PS 38:3(18:0/20:3)	-1.56	0.0010	FFA22:6	-1.13	0.0023

^a^Fold changes (FC) were calculated as the average levels in the P + 21 d (d 21postpartum) group relative to those in the P 0 d (the day of kidding) group. The Log2FC greater than 0 indicates a relatively higher concentration in the P + 21 d group, whereas the Log2FC of less than 0 indicates a concentration lower than that in the P 0 d group. FDR, false discovery rate

^b^*Cer* Ceramides, *LPI* Lyso-phosphatidylinositols, *PA* Phosphatidic acids, *PC* Phosphatidylcholines, *TAG* Triacylglycerols, *SM* Sphingomyelins, *PE* Phosphatidylethanolamines, *PS* Phosphatidylserines, *FFA* Free fatty acids

**Fig. 7:**
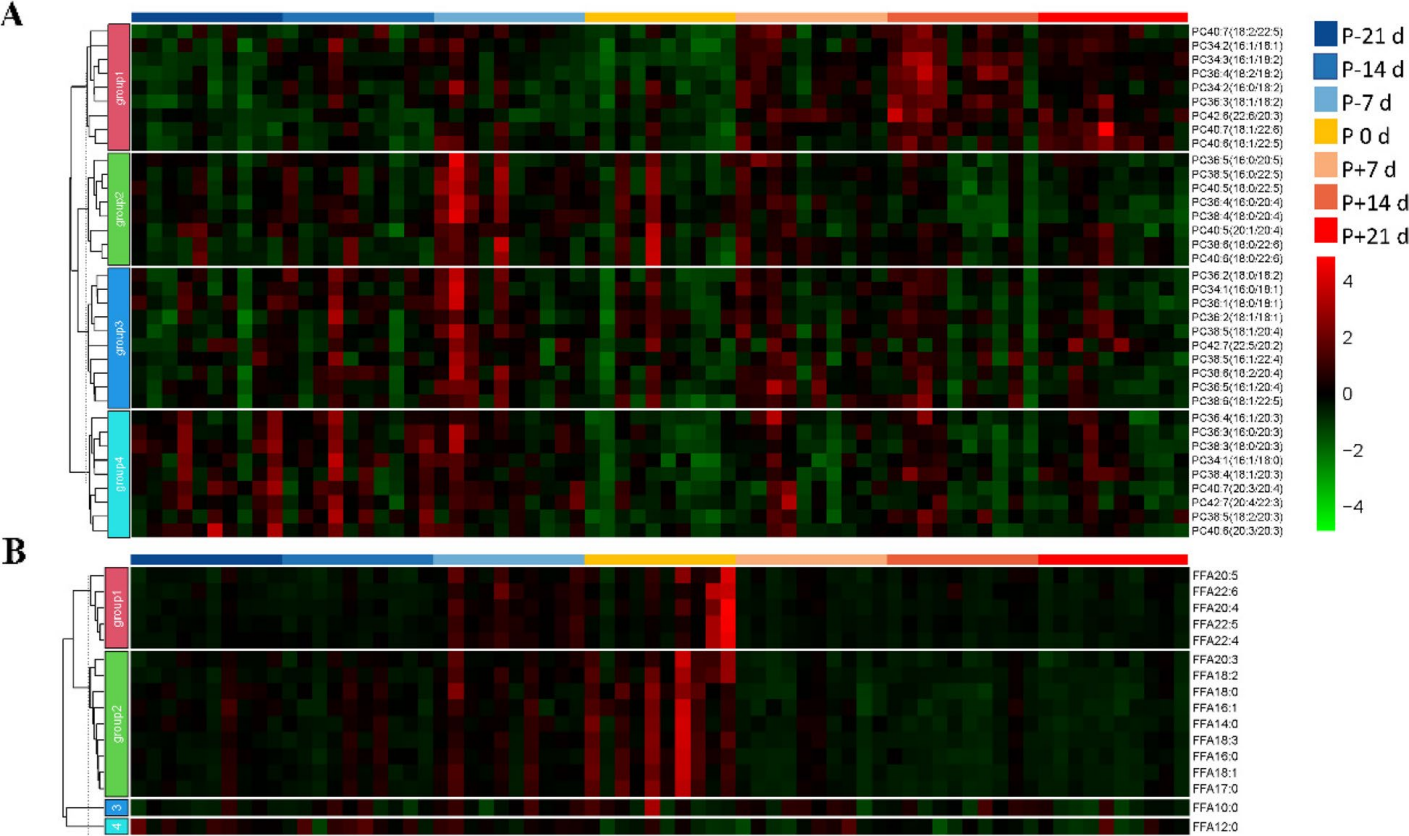
Hierarchical clustering heatmap of the phosphatidylcholines (PC, **A**) and free fatty acids (FFA, **B**) in plasma. P-21 d, P-14 d, P-7 d (d 21,14 and 7 before the due date), P 0 d (the day of kidding), and P + 7 d, P + 14 d, P + 21 d (d 7, 14, and 21postpartum)

**Fig. 8:**
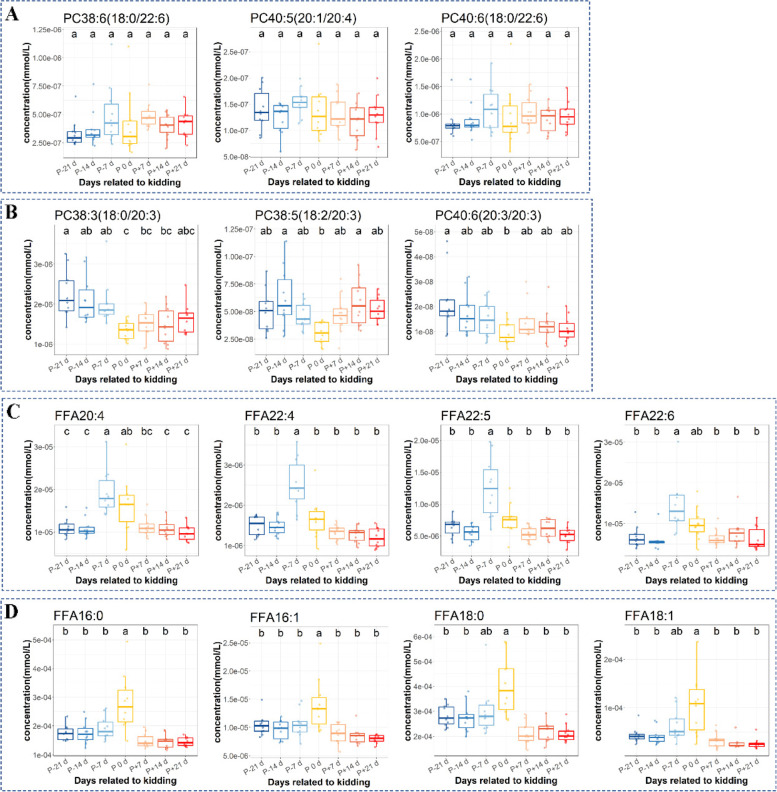
Peripartal changes in phosphatidylcholines (PC) and free fatty acids (FFA) with differences in carbon chain saturation and length: **A** PC with 22:6 and 20:4 carbon chains, **B** PC with 22:3 carbon chains, **C** PUFA, **D** SFA and MUFA. Mean values with different letters (a–c) show statistically significant differences (LSD, *P* < 0.05). P-21 d, P-14 d, P-7 d (d 21,14 and 7 before the due date), P 0 d (the day of kidding), and P + 7 d, P + 14 d, P + 21 d (d 7, 14, and 21postpartum)

Hierarchical clustering heatmap plots for PC and FFA were generated using hierarchical clustering analysis (Fig. 7). PC38:6(18:0/22:6), PC40:6(18:0/22:6), PC40:5(20:1/20:4) were clustered together (Fig. 7A, group2) and did not change significantly in the peripartal period (Fig. 8A). However, the PC species with fatty acyl chains of 20:3 (Fig. 7A, group4) were clustered on the other side and decreased significantly at P 0 d (Fig. 8B). The polyunsaturated fatty acids (PUFA) with more than 20 carbons and other fatty acids were divided into two categories using hierarchical clustering (Fig. 7B, group1, and group2). The PUFA (C20:4, C22:4, C22:5, C22:6) increased at P-7 d, and then declined or remained unchanged at P 0 d (Fig. 8C). The SFA (saturated fatty acid, C16:0, C18:0) and MUFA (monounsaturated fatty acid, C16:1, C18:1) were significantly elevated at P 0 d (Fig. 8D). Lastly, there were interrelationships among the differential metabolites, screened by lipidomics and untargeted metabolomics, and biochemical markers (Fig. S4).

## Discussion

The untargeted metabolomics measurements revealed that metabolic profiles of goats in the antepartum and postpartal periods were different from those on the day of kidding. The resilience to metabolic stress is crucial for determining subsequent health, production, and reproductive performance (Matthews 2016). Thus, the present study aimed to investigate the changes and interactions of various small-molecular-weight metabolites in the plasma of dairy goats during the peripartal period.

Fatty acids derived from adipose tissue lipolysis are oxidized for providing energy primarily through mitochondrial β-oxidation. An increase in fatty acid oxidation induces excessive oxidative stress, and the observed increases in 2-hydroxybutyrate and uric acid reflected an imbalance between prooxidants and antioxidants (Ames et al. 1981; Gall et al. 2010; Fahrmann et al. 2015). Our previous study demonstrated a sustained state of oxidative stress in dairy goats during the peripartal period (Huang et al. 2021). Oxidative stress has the potential to restrict the functioning of α-ketoglutarate dehydrogenase in mitochondria, thus resulting in the accumulation of α-ketoglutarate (Tretter and Adam-Vizi 2005). Uric acid inhibits mitochondrial aconitase activity, but activates ATP citrate lyase (Lanaspa et al. 2012). A reduction in the activity of aconitase inhibits the formation of citric acid, leading to lower citric acid on the day of kidding. As important intermediates of the TCA cycle, changes in α-ketoglutarate and citric acid indicate TCA cycle flow interruption. A study of clinical ketosis in cows demonstrated that defects in TCA cycle flux can aggravate the severity of ketosis (Zhang et al. 2013). Therefore, the increase in the capacity of the TCA cycle is important to prevent energy metabolism disorders in transition dairy goats. Pyruvate not only enters the TCA cycle through the action of pyruvate dehydrogenase, but also generates glucose through the action of pyruvate carboxylase and phosphoenolpyruvate carboxylase (PEPCK). Alterations in the TCA cycle could account for the accumulation of pyruvate, channeling it to the gluconeogenesis pathway (Yoshimi et al. 2016). Greenfield et al. (Greenfield et al. 2000) speculated that an increase in abundance of pyruvate carboxylase and PEPCK mRNA during the early transition period was indicative of an increase in gluconeogenesis. The plasma glucose concentration during lactation was greater compared with late-pregnancy in Saanen goats (Sadjadian et al. 2013). Thus, alterations in the TCA cycle support gluconeogenesis during lactation in dairy goats.

To date, there is very little published information on amino acid metabolism in dairy goats during the transition period. In present sudy, multiple amino acid levels decreased after calving. The decrease in some amino acids (Trp, Tyr, and Val) can profoundly affect the overall rate of protein synthesis and contribute to lower plasma TP especially in the postpartum. Furthermore, some studies reported that fluctuations in the plasma amino acid levels are associated with ketosis in dairy cows (Zhang et al. 2013; Li et al. 2014). Changes in phenylacetylglycine and hippuric acid levels on the phenylalanine metabolic pathway were observed. Both the aromatic amino acids Trp and Tyr were also reduced on the day of kidding. All these observations point to the perturbation of phenylalanine metabolism. Phenylalanine is metabolized to tyrosine by phenylalanine hydroxylase and is involved in synthesizing various hormones (thyroid hormone, melanin, catecholamine, epinephrine, norepinephrine, and dopamine) and glycolipid metabolism (Lemmon and Schlessinger 2010). Deficiencies of Phe and Tyr reportedly impaired immune responses in chickens; Dietary supplementation of amino acids could enhance the immune response (Konashi et al. 2000). Changes in the phenylacetylglycine levels are associated with disorders of phospholipid metabolism (Delaney et al. 2004). The ratio of phenylacetylglycine to hippuric acid can serve as a biomarker for phospholipid disease (Kamiguchi et al. 2017; Malek et al. 2020). The increase in phenylacetylglycine and the decrease in hippuric after parturition in the present study suggested the possibility of altered phospholipid metabolism in dairy goats. Because phospholipids are important lipids involved in the cellular inflammatory response and immune regulation, the observed changes could be important to understand the occurrence and development of inflammation and disease in transition goats.

Indole sulfate (IS) and p-cresol sulfate (PCS) are metabolites of aromatic amino acids originating from protein fermentation in the intestine (Evenepoel et al. 2009; Opdebeeck et al. 2020). In clinical investigations, IS and PCS have been found to induce vascular toxicity followed by upregulation of inflammatory, coagulation, and oxidative stress pathways (Opdebeeck et al. 2020). There is a report that PCS in urine can predict nitrogen intake and efficiency of use (Bertram et al. 2011). Thus, the increase in the IS and PCS in early lactation might indicate a similar involvement for these compounds.

Phosphatidic acids, a central intermediate in synthesizing PS, PC, PE, and PI (Coleman and Mashek 2011), was reduced by nearly 40% on the day of kidding. The quantity of glycerophospholipids particularly PS and PE, which are important components of biofilm, were elevated postpartum (Cole et al. 2012). Multiple studies have shown that lysophospholipid (LPL), one of the phospholipid metabolites, is associated with fatty liver, steatohepatitis, diabetes, obesity, and even cancer (Grzelczyk and Gendaszewska-Darmach 2013). Thus, elevated levels of LPL in this study suggested a similar involvement in the peripartal period. Phosphatidic acid is hydrolyzed by phosphatidic acid phosphatase to DAG, the penultimate step in TAG biosynthesis (Coleman and Mashek 2011). An increased use of PA for synthesis of DAG and TAG might account for the decrease in PA and LPA at the day of kidding.

It is known that the changes of FFA were significant during the peripartal period. However, it was noteworthy that the distinctions have been identified with regards to alterations in SFA and PUFA. Whereas SFA are considered to negatively affect human health (Hu et al. 2001), PUFA are linked to anti-inflammatory events (Calder 2006) as well as improved immune system function (Srednicka-Tober et al. 2016). Supplemental PUFA in goat diets increases the number of follicles and ovulation rate, shortens the estrus cycles, and improves the immune response (Agazzi et al. 2004; Mahla et al. 2017; Stergiadis et al. 2019) Goat milk contains higher levels of PUFA compared to cow milk (Stergiadis et al. 2019). Hence, initiating lactation may be responsible for part of the lowered PUFA at the day of kidding. We observed that oleic acid and linoleic acid levels in the plasma increased significantly at the day of kidding. Consistently, in a study with dairy cows, the oleic acid levels were found to increase at the day of calving and in individuals with subclinical mastitis (Dervishi et al. 2016; Luo et al. 2019). Rukkwamsuk et al. (1999) found that cows on high-energy diet experienced a deeper negative energy balance compared to controls, and also showed a higher proportion of oleic acid in the plasma NEFA after parturition. Thus, dietary supplements rich in PUFA may be a better choice for dairy goats after parturition.

Carnitine is converted to ACs when fatty acids are shuttled into the mitochondria via carnitine palmitoyltransferase-1 (McGarry and Brown 1997). Increased fatty acid oxidation in liver and skeletal muscle is one of the reasons for carnitine's continued decline during the perinatal period (Krajcovicova-Kudlackova et al. 2000; Yang et al. 2019). Correspondingly, the level of ACs will increase (Ismaeel et al. 2019; Yang et al. 2019). We have focused on the long-chain ACs (C16, C17, C18) as it is associated with the increased fatty acid load (Schooneman et al. 2013; McFadden 2020; Schren et al. 2021). Rapidly changing fatty acid and ACs levels reflect the high energy demands during parturition. Neutral lipid depots are mobilized to provide energy for parturition, which perhaps saturates the mitochondrial capacity to cope with the fatty acid surplus and results in the temporal elevation of circulating ACs, dropping to the prenatal level soon after parturition.

Peripartal changes in the circulating NEFA and BHB have been reported in Surti goats and Saanen goats (Sadjadian et al. 2013; Manat et al. 2016; Huang et al. 2021). A rapid drop in NEFA levels at P + 7 d was also observed in dairy goats with a high energy diet (Celi et al. 2008). Circulating ACs and NEFA levels are rapidly reduced postpartum, suggesting that both the production and oxidation of NEFA were reduced postpartum in present study. Thus, increasing the dry matter intake of dairy goats during the peripartal period can effectively reduce the impact of negative energy balance.

Excess fatty acids promote the increase in TAG synthesis in the liver, known as re-esterification. The export of this part of the TAG from the liver depends on very-low-density lipoproteins (VLDL). The ability of the liver to synthesize cholesterol is also significantly increased in early lactation to ensure the VLDL synthesis (Schlegel et al. 2012; Kessler et al. 2014). Cholesterol and TAG synthesized by the liver are secreted into the circulation as VLDL. In ruminants, however, the ability of the liver to assemble and secrete VLDL is inherently lower than that in other mammals, and the accumulation of free fatty acids further impairs VLDL secretion (Bobe et al. 2004). Thus, the reduction in postpartum plasma TAG and CE might be due to restricted hepatic VLDL export and an increase in the clearance of circulating TAG by lipoprotein lipase in the mammary gland (Zang et al. 2019). The low levels of TAG and CE were maintained until the end of the experiment, which reflected the rate of VLDL secretion from liver maintained at lower levels, similar to a study in dairy cows (Van den Top et al. 2005; Kessler et al. 2014). Multiple studies in dairy cows have demonstrated that reduced hepatic TAG output leads to TAG accumulation, especially in cows with ketosis (Gross et al. 2013; Zang et al. 2019; Vogel et al. 2020).

Phosphatidylcholines is a major structural component of the cellular membranes, and is essential for synthesizing VLDL (Yao and Vance 1988; McFadden et al. 2020). Betaine and methionine drive the production of the universal methyl donor S-adenosylmethionine, supporting the production of PC through the phosphatidylethanolamine N-methyltransferase (PEMT) pathway (Pinotti et al. 2002). In addition, PC can be synthesized from choline through the cytidine-5′-diphosphate (CDP) choline pathway (McFadden et al. 2020). In nonruminants, Zeisel (Zeisel 1992) suggested choline deficiency (particularly PC) as the main reason for impaired secretion of VLDL. In some instances, supplementation with dietary methyl donors reduces lipid accumulation in the liver of dairy cows (Zang et al. 2019). Choline is an important methyl donor, existing in mammals in various forms such as acetylcholine, betaine, methionine, phosphatidylcholine, and sphingomyelin. Early lactating cows have a high demand for methyl compounds, but exogenous methyl donor supply is reduced due to lower DMI and extensive rumen degradation (Xue and Snoswell 1986). Hence, ruminants must adapt their metabolism of methyl groups during the transition into lactation. In this study, betaine (a product of choline oxidation) and methionine were observed to be at their highest levels around kidding. Feeding rumen-protected choline and methionine improves metabolic status, reduces oxidative stress, and enhances immune function (Shahsavari et al. 2016; Zhou et al. 2016; Batistel et al. 2018). Moreover, betaine-supplemented diets tend to improve production performance of dairy cows (Monteiro et al. 2017).

Mouse studies show that the CDP-choline pathway is biased toward the use of DAG-rich saturated or monounsaturated fatty acids (i.e., palmitic and oleic acids, respectively) as substrates; In contrast, the PEMT pathway prefers the use of long-chain and very-long-chain rich phosphatidylethanolamines of the PUFA as substrates, including eicosatetraenoic acid and docosahexaenoic acid (DHA) (DeLong et al. 1999). The circulating PC containing DHA has been identified as a biomarker of PEMT pathway activation in humans (da Costa et al. 2011). Our results suggest that inhibition of the CDP-choline pathway might be caused by choline deficiency and a compensatory increase of the PEMT pathway in early lactation. Quantification of VLDL secretion from primary bovine hepatocytes by ELISA showed that the VLDL output increased with choline supplementation (Chandler and White 2017). Cows with severe hepatic steatosis have lower serum PC levels compared to the clinically healthy cows (Imhasly et al. 2014). Thus, considering that the changes in choline metabolism might reflect the state of hepatic lipid metabolism in dairy goats, it can be suggested that dietary choline supplementation might reduce the pressure of hepatic lipid accumulation.

Circulating Cer levels were elevated postpartum, especially Cer(d18:0/25:1). Similar results of increased Cer levels in the transition from gestation to lactation were observed in dairy cows (Rico et al. 2015). Elevated Cer might be associated with hepatic fat accumulation, inflammation, and hydrolysis of SM (Peraldi et al. 1996; Rico et al. 2018). More interestingly, the increase in Cer often coincides with the decrease in insulin sensitivity (Rico et al. 2015). During early lactation, insulin resistance was found to enhance adipose tissue mobilization and promote the preferential allocation of glucose to the mammary gland, thereby increasing the synthesis of milk fat and lactose and milk production (Bell 1995; Zachut et al. 2013; Rico et al. 2015). However, there are no previous studies that have clarified the role of Cer(d18:0/25:1) in peripartal ruminants, limiting our understanding of the bioactivity of Cer(d18:0/25:1).

## Materials and methods

### Animals and study design

The experiments were conducted in accordance with the university’s guidelines for animal research at the experimental farm of Northwest A&F University (Shaanxi Province, China) in Western China (106°55′57″E, 34°48′41″N) in January–March 2019.

Ninety-six primiparous Guanzhong dairy goats (ranging in age from 1 to 2 years; body weight: 60 ± 5.2 kg; DMI: 1.45 ± 0.05 kg/d; mean ± standard deviation) were used as initial experimental animals. Goats were housed in a shaded open barn under natural lighting conditions and had free access to freshwater. Goats were fed the same diet offered twice daily at 0730 and 1530 ad libitum as a TMR. Diets were formulated to meet nutrient requirements of dairy goats according to the Nutrient Requirements of Small Ruminants (National Research Council, 2007). Ingredients of the diets are listed in Table 5.

**Table 5:** Ingredients and chemical composition of the antepartum and postpartum diets on a DM basis^a^

Ingredient (% of DM)	Antepartum	Postpartum	Nutrient composition	Antepartum	Postpartum
Alfalfa hay	15.36	18.42	DM (% of fresh)	45.10	48.20
Corn	20.72	23.16	Neutral detergent fiber (NDF, %)	42.20	37.70
Wheat bran	7.20	8.37	Acid detergent fiber (ADF, %)	18.50	17.40
Soybean meal	4.89	9.02	Crude protein (CP, %)	14.20	16.40
Wheat straw	7.57	0.00	Starch (%)	24.3	25.40
Corn Silage	35.35	30.66	Ether extract (%)	3.00	3.20
Corn germ meal	2.80	3.26	Calcium (Ca, %)	0.48	0.66
Cottonseed meal	4.40	5.12	Phosphorus (P, %)	0.36	0.37
Calcium hydrophosphate	0.44	0.51	Magnesium (Mg, %)	0.14	0.19
Limestone	0.40	0.46	Sulfur (S, %)	0.20	0.20
Sodium carbonate	0.32	0.38	Chloride (Cl, %)	0.47	0.25
Sodium chloride	0.40	0.45	DCAD (mEq/kg of DM)	-164	+ 733
Mineral and vitamin premix^a^	0.15	0.19	NE_L_ (Mcal/kg)	1.53	1.62

^a^The mineral-vitamin premix provided the following per kg of diets: vitamin A 250,000 IU, vitamin D 23,250 IU, vitamin E 1500 IU, manganese 800 mg, zinc 1800 mg, copper 370 mg, iron 2200 mg, cobalt 50 mg, iodine 30 mg, selenium 30 mg

Notably, in September, the farm goats included in this study were estrous synchronized such that kidding occurred in February. Blood samples collection was performed every other week from 21 d before kidding (22 to 20 d before the expected kidding date0) to 21 d postpartum. The subset of goats used was selected to include only healthy animals with a gap between expected and real kidding day of two days or less. Additionally, 7 goats were excluded for their litter size being 2 or 3. Finally, the final experimental sample size was 9. Over the period of the trial, the average milk yield per dairy goat was 1.8 ± 0.3 kg/d (mean ± standard deviation). The samples were divided into 7 groups according to sampling time: d 21,14 and 7 before due date (P-21 d, P-14 d, P-7 d), the day of kidding (P 0 d), and d 7, 14, and 21 postpartum (P + 7 d, P + 14 d, P + 21 d).

### Blood samples collection

Blood was collected (10 mL) at 0600 h over seven time points (d 21, 14, 7 before and d 7, 14, 21 postpartum). In addition, Sample at P 0 d was collected within 1 h after kidding. Plasma was collected using EDTA as an anticoagulant, then centrifuged at 1,500 × g for 10 min at 4℃, and subsequently stored at − 80 °C until analysis.

### Biochemical analyses

The concentrations of GLU, aminotransferase (AST), alanine aminotransferase (ALT), cholinesterase (CHE), γ-glutamyl transpeptidase (GGT), lactate dehydrogenase (LDH), alkaline phosphatase (ALP), total protein (TP), albumin (ALB), high-density cholesterol (HDL), low-density cholesterol (LDL), triglyceride (TG), total cholesterol (TC), urea in plasma were determined with a chemical autoanalyzer (Hitachi 7060, Hitachi, Tokyo).

The levels of non–esterified fatty acids (NEFA, kit no. FA115, enzymatic method) and β-hydroxybutyrate (BHB, kit no. RB1007, colorimetric method) were determined with the commercial kits (Randox Laboratories, Crumlin, UK).

### Metabolome extraction

Samples were prepared according to a previous method (Song et al. 2020). Briefly, a 50 µL sample was mixed with 200 µL ice-cold 80% methanol in water, and incubated for 30 min at 1500 rpm and 4℃ followed by centrifugation for 10 min at 16,260 × g and 4℃. The supernatant was then removed into a clean 1.5 mL centrifuge tube, and dried using a SpeedVac. The dried extracts were redissolved with 1% acetonitrile in water, and the liquid in the upper layer was collected for liquid chromatography triple quadrupole mass spectrometry (LC–MS) analysis.

### Untargeted metabolomics analysis

Untargeted metabolomics was conducted by LipidALL Technologies as previously described (Song et al. 2020). Metabolites were separated on an ACQUITY UPLC HSS T3 1.8 μm, 2.1 × 100 mm column (Waters, Dublin, Ireland) using ultra-performance liquid chromatography (Agilent 1290 II, Agilent Technologies, Germany) and analyzed on a Quadrupole-TOF MS (5600 Triple TOF Plus, AB SCIEX, Singapore). The MS parameters for detection were: ESI source voltage − 4.5 kV; vaporizer temperature, 500℃; drying gas (N_2_) pressure, 50 psi; nebulizer gas (N_2_) pressure, 50 psi; curtain gas (N_2_) pressure, 35 psi; the scan range was m/z 60–800. The information-dependent acquisition mode was used for MS/MS analyses of the metabolites. The collision energy was set at 35 ± 15 eV and the data acquisition and processing were performed using the Analyst® TF 1.7.1 Software (AB Sciex, Concord, ON, Canada). All detected ions were extracted using MarkerView 1.3 (AB Sciex, Concord, ON, Canada) into Excel in the format of a two-dimensional matrix, including mass to charge ratio (m/z), retention time, and peak areas. Then, the isotopic peaks were filtered. The MS/MS data were extracted, and a comparison was performed with the Metabolites database (AB Sciex, Concord, ON, Canada), HMDB, METLIN using the PeakView 2.2 (AB Sciex, Concord, ON, Canada), and the standard references were used for annotating the ion ID.

### Targeted lipidomics analysis

Lipids were extracted from the plasma (20 µL) using a modified Bligh and Dyer’s extraction procedure (double rounds of extraction) and dried in a SpeedVac under OH mode (Song et al. 2020). Before analysis, the lipid extracts were resuspended in chloroform: methanol 1:1 (v/v) spiked with appropriate internal standards. The lipidomic analyses was carried out on an Exion UPLC system coupled with a QTRAP 6500 PLUS system (Sciex) under an electrospray ionization mode as described previously unless otherwise stated (Lam et al. 2021). All the quantification experiments were conducted using an internal standard calibration. The lipid types measured include, acylcarnitines (ACs), cholesteryl esters (CE); ceramides (Cer); diacylglycerols (DAG); free fatty acids (FFA); monosialogangliosides (GM3); phosphatidic acids (PA); phosphatidylcholines (PC); lyso-PC (LPC); phosphatidylethanolamines (PE); phosphatidylglycerols (PG); phosphatidylinositols (PI); phosphatidylserines (PS); sphingosine-1-phosphate (S1P); sphingomyelins (SM); triacylglycerols (TAG).

In brief, the polar lipids were separated on a Phenomenex Luna Silica 3 µm column (i.d. 150 × 2.0 mm) using the mobile phase A (chloroform: methanol: ammonium hydroxide, 89.5:10:0.5) and mobile phase B (chloroform: methanol: ammonium hydroxide: water, 55:39:0.5:5.5) at a flow rate of 270 µL/min and column oven temperature at 25℃. The individual polar lipid species were quantified by referencing to spiked internal standards including PC-14:0/14:0,PE14:0/14:0, d31-PS-16:0/18:1, PS-17:0/20:4, PA-17:0/17:0, PG-14:0/14:0,GluCer-d18:1/8:0, Cer-d18:1/17:0, C14:0-BMP, S1P-d17:1, Sph-d17:1,SM-d18:1/12:0, LPC-17:0, LPE-17:1, LPI-17:1, LPA-17:0, LPS-17:1 obtained from Avanti Polar Lipids and PI-8:0/8:0 from Echelon Biosciences, Inc. The monosialogangliosides (GM3) species were quantified using GM3d18:1/18:0-d3 from Matreya LLC.

The glycerol lipids including DAG and TAG were quantified using a modified version of reverse phase HPLC/MRM (Song et al. 2020). Neutral lipids were separated on a Phenomenex Kinetex-C18 2.6 µm column (i.d. 4.6 × 100 mm) using an isocratic mobile phase containing chloroform: methanol:0.1 M ammonium acetate 100:100:4 (v/v/v). Levels of short-, medium-, and long-chain TAGs were calculated with reference to the spiked internal standards of TAG(14:0)3-d5, TAG(16:0)3-d5, and TAG(18:0)3-d5 obtained from the CDN isotopes, respectively. The DAG were quantified using the d5-DAG16:0/16:0 and d5-DAG18:1/18:1as internal standards (Avanti Polar Lipids). The free cholesterols and CE were quantitated with d6-cholesterol and d6-C18:0 cholesteryl ester (CDN isotopes) as the internal standards under atmospheric pressure chemical ionization. Plasma lipid levels are expressed in nanomoles per L (nmol/L).

### Data processing and statistical analyses

Data obtained from the biochemical analyses were statistically analyzed using GraphPad Prism 8.0 (GraphPad Software Inc., USA). Changes in the data from the biochemical analysis were analyzed using repeated measures ANOVA followed by Tukey’s multiple comparisons test. The repeated measures on each goat were considered (repeated factor: time during the peripartal period) and the results were expressed as means ± SEM.

Untargeted metabolomics data were pareto-scaled and pattern recognition analysis was performed using the SIMCA-P software (version 14.1, Umetrics, Umea, Sweden), comprising the unsupervised principal component analysis (PCA) and the supervised orthogonal partial least squares discriminant analysis (OPLS-DA). The intra-group aggregation and inter-group separation tendencies were determined using PCA and the inter-group differences were further determined using OPLS-DA. The OPLS-DA models were validated based on the interpretation of variation in Y (R^2^Y) and the forecast ability based on the model (Q^2^) in cross-validation and permutation tests by applying 200 iterations. The differential metabolites were screened using variable importance in projection (VIP) scores (VIP > 1) obtained from the OPLS-DA model and *P*-values (*P* < 0.05) from the paired t-test. Pathway analyses were performed using MetaboAnalyst 4.0 (http://www.metaboanalyst.ca) and the clustering was further performed using the R heatmap package (version 4.1.3, http://www.R-project.org).

Lipidomics data were analyzed in R using the Limma statistical package. The limma model compared the variation in lipidome within-subjects over time by treating the goats as random effect and estimated the correlation between measurements made on the same goat. The differentially-expressed lipids based on false discovery rate (FDR) and fold-change (FC) were considered statistically significant (FDR < 0.05 and |log2FC|> 1). A volcano plot was used for visualizing the differentially-expressed lipids. The boxplot was generated via the boxplot function in R and the differences was analyzed using R (LSD.test function in the “agricolae” package). A correlation matrix was produced using the corrplot function with the R package. To avoid false positives, correlations with adjusted *P* < 0.01 were selected. For visual simplicity, only significant correlations were shown.

## Supplementary Information

**Additional file 1: Fig. S1.** Metabolite profiles of peripartal dairy goats: (A and B) orthogonal partial least squares discriminant analysis (OPLS-DA) score plot and permutation test plots for P-14 d vs. P 0 d, (C and D) OPLS-DA score plot and permutation test plots for P-7 d vs. P 0 d. P 0 d, (E and F) OPLS-DA score plot and permutation test plots for P 0 d vs. P+7d. P 0 d, (G and H) OPLS-DA score plot and permutation test plots for P 0 d vs. P+14d. t[1] = first principal component. to[2] = second orthogonal component. The intercept limit of Q^2^, calculated by the regression line, is the plot of Q^2^ from the permutation test in the OPLS-DA model. P-14 d, P-7 d (d 14 and 7 before the due date), P 0 d (the day of kidding), and P+7 d, P+14 d (d 7 and 14 postpartum). **Fig. S2.** Box-plot (middle bar = median, box limit = upper and lower quartile, extremes = Min and Max values) depicting the peripartal changes in differentially altered metabolites detected via non-targeted metabolomics. P-21 d, P-14 d, P-7 d (d 21,14 and 7 before the due date), P 0 d (the day of kidding), and P+7 d, P+14 d, P+21 d (d 7, 14, and 21postpartum). Mean values with different letters (a–d) show statistically significant differences based on least significant difference (LSD) (*P* < 0.05). **Fig. S3.** Line graph depicting the peripartal changes for all lipid species levels in dairy goats. P-21 d, P-14 d, P-7 d (d 21,14 and 7 before the due date), P 0 d (the day of kidding), and P+7 d, P+14 d, P+21 d (d 7, 14, and 21postpartum). Results are expressed as means ± SEM. ACs = acylcarnitine; CE = cholesteryl esters; Cer = ceramides; DAG = diacylglycerols; FFA = free fatty acids; GM3 = monosialogangliosides; PA = phosphatidic acids; PC = phosphatidylcholines; LPC = lyso-PC; PE = phosphatidylethanolamines; PG = phosphatidylglycerols; PI = phosphatidylinositols; PS = phosphatidylserines; S1P = sphingosine-1-phosphate; SM = sphingomyelins; TAG = triacylglycerols. **Fig. S4.** Correlations between plasma biochemical indices, differentially altered metabolites, and lipid levels at all time points combined. The left margin shows a dendrogram from hierarchical cluster analysis by which rows and columns are ordered.

## Data Availability

The data and materials that support the findings of this study are available from the corresponding author upon request.

## Abbreviations

NEB: Negative energy balance
GLU: Glucose
ACs: Acylcarnitines
AST: Aminotransferase
ALT: Alanine aminotransferase
CHE: Cholinesterase
GGT: γ-Glutamyl transpeptidase
LDH: Lactate dehydrogenase
ALP: Alkaline phosphatase
TP: Total protein
ALB: Albumin
HDL: High-density cholesterol
LDL: Low-density cholesterol
TG: Triglyceride
TC: Total cholesterol
NEFA: Non–esterified fatty acids
BHB: β-Hydroxybutyrate
CE: Cholesteryl esters
Cer: Ceramides
DAG: Diacylglycerols
FFA: Free fatty acids
GM3: Monosialogangliosides
PA: Phosphatidic acids
PC: Phosphatidylcholines
LPC: Lyso-PC
PE: Phosphatidylethanolamines
PG: Phosphatidylglycerols
PI: Phosphatidylinositols
PS: Phosphatidylserines
S1P: Sphingosine-1-phosphate
SM: Sphingomyelins
TAG: Triacylglycerols
PCA: Principal component analysis
OPLS-DA: Orthogonal partial least squares discriminant analysis
VIP: Variable importance in projection
FDR: False discovery rate
FC: Fold-change
PEPCK: Phosphoenolpyruvate carboxylase
IS: Indole sulfate
PCS: P-cresol sulfate
LPL: Lysophospholipid
VLDL: Very-low-density lipoproteins
PEMT: Phosphatidylethanolamine N-methyltransferase
CDP: Cytidine-5′-diphosphate
DHA: Docosahexaenoic acid
SFA: Saturated fatty acid
MUFA: Monounsaturated fatty acid
PUFA: Polyunsaturated fatty acids
