# Supplementary material for: Effects of perinatal stress on the metabolites and lipids in plasma of dairy goats
Source: Stress Biol. 2023 May 12;3(1):11. doi: 10.1007/s44154-023-00088-z (PMC10441998; doi:10.1007/s44154-023-00088-z)
Supplement: Supplementary file 1 — Additional file 1: Fig. S1. Metabolite profiles of peripartal dairy goats: (A and B) orthogonal partial least squares discriminant analysis (OPLS-DA) score plot and permutation test plots for P-14 d vs. P 0 d, (C and D) OPLS-DA score plot and permutation test plots for P-7 d vs. P 0 d. P 0 d, (E and F) OPLS-DA score plot and permutation test plots for P 0 d vs. P+7d. P 0 d, (G and H) OPLS-DA score plot and permutation test plots for P 0 d vs. P+14d. t[1] = first principal component. to[2] = second orthogonal component. The intercept limit of Q2, calculated by the regression line, is the plot of Q2 from the permutation test in the OPLS-DA model. P-14 d, P-7 d (d 14 and 7 before the due date), P 0 d (the day of kidding), and P+7 d, P+14 d (d 7 and 14 postpartum). Fig. S2. Box-plot (middle bar = median, box limit = upper and lower quartile, extremes = Min and Max values) depicting the peripartal changes in differentially altered metabolites detected via non-targeted metabolomics. P-21 d, P-14 d, P-7 d (d 21,14 and 7 before the due date), P 0 d (the day of kidding), and P+7 d, P+14 d, P+21 d (d 7, 14, and 21postpartum). Mean values with different letters (a–d) show statistically significant differences based on least significant difference (LSD) (P < 0.05). Fig. S3. Line graph depicting the peripartal changes for all lipid species levels in dairy goats. P-21 d, P-14 d, P-7 d (d 21,14 and 7 before the due date), P 0 d (the day of kidding), and P+7 d, P+14 d, P+21 d (d 7, 14, and 21postpartum). Results are expressed as means ± SEM. ACs = acylcarnitine; CE = cholesteryl esters; Cer = ceramides; DAG = diacylglycerols; FFA = free fatty acids; GM3 = monosialogangliosides; PA = phosphatidic acids; PC = phosphatidylcholines; LPC = lyso-PC; PE = phosphatidylethanolamines; PG = phosphatidylglycerols; PI = phosphatidylinositols; PS = phosphatidylserines; S1P = sphingosine-1-phosphate; SM = sphingomyelins; TAG = triacylglycerols. Fig. S4. Correlations between plasma biochemical ind [file 44154_2023_88_MOESM1_ESM.docx]

**Supplementary Material**


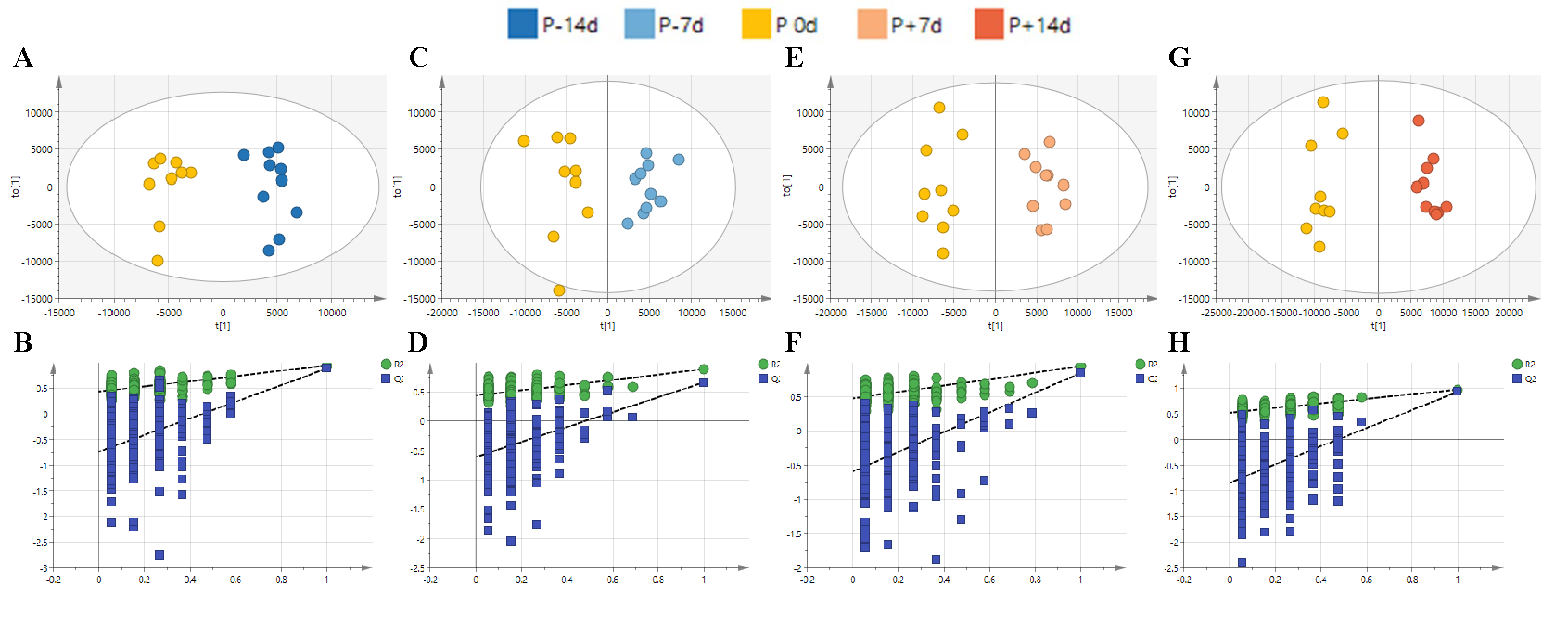
 **Fig. S1** Metabolite profiles of peripartal dairy goats: (A and B) orthogonal partial least squares discriminant analysis (OPLS-DA) score plot and permutation test plots for P-14 d vs. P 0 d, (C and D) OPLS-DA score plot and permutation test plots for P-7 d vs. P 0 d. P 0 d, (E and F) OPLS-DA score plot and permutation test plots for P 0 d vs. P+7d. P 0 d, (G and H) OPLS-DA score plot and permutation test plots for P 0 d vs. P+14d. t[1] = first principal component. to[2] = second orthogonal component. The intercept limit of Q^2^, calculated by the regression line, is the plot of Q^2^ from the permutation test in the OPLS-DA model. P-14 d, P-7 d (d 14 and 7 before the due date), P 0 d (the day of kidding), and P+7 d, P+14 d (d 7 and 14 postpartum).


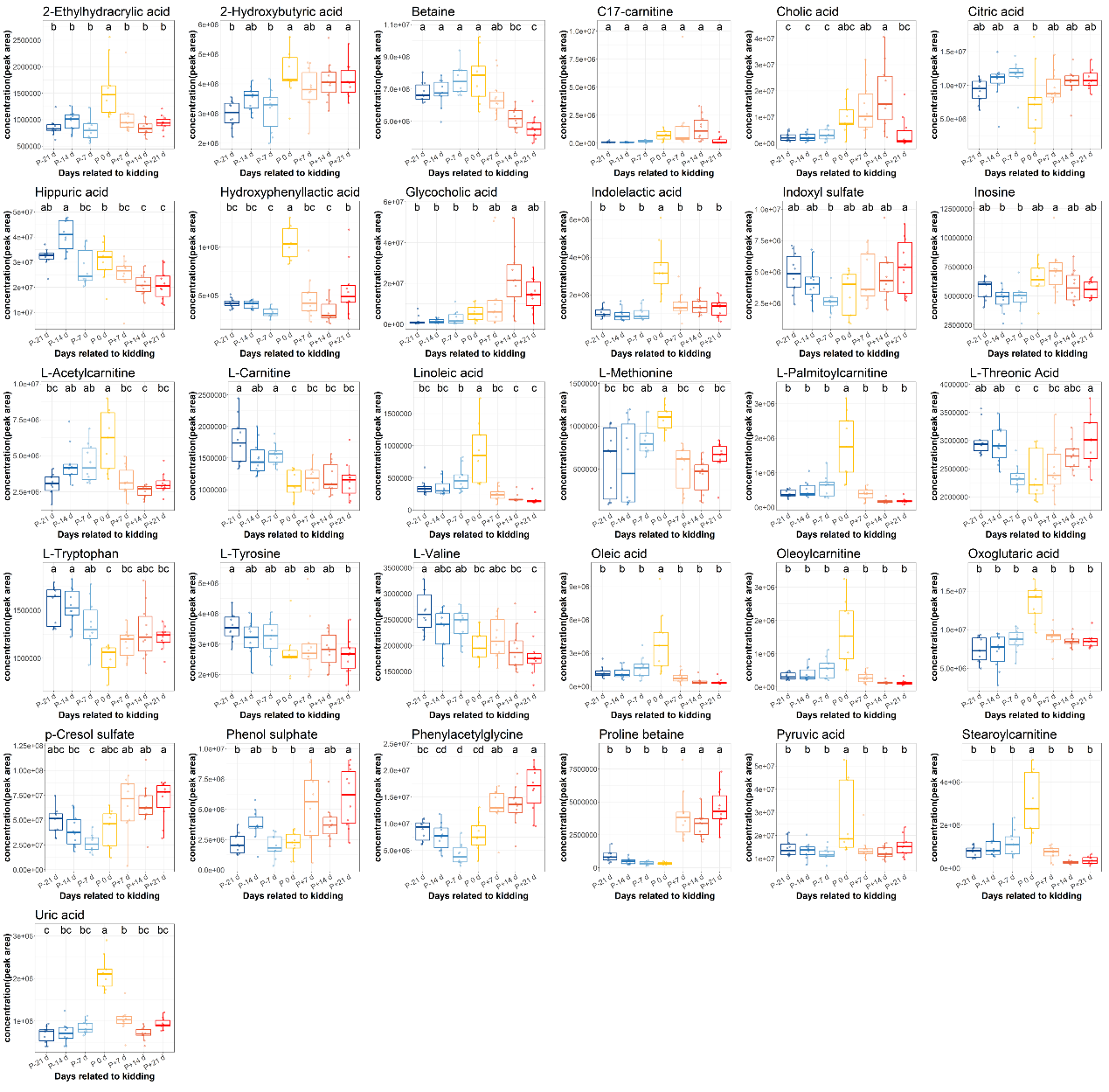
 **Fig. S2** Box-plot (middle bar = median, box limit = upper and lower quartile, extremes = Min and Max values) depicting the peripartal changes in differentially altered metabolites detected via non-targeted metabolomics. **P-21 d**, **P-14 d**, **P-7 d** (d 21,14 and 7 before the due date), **P 0 d** (the day of kidding), and **P+7 d**, **P+14 d**, **P+21 d** (d 7, 14, and 21postpartum). Mean values with different letters (a–d) show statistically significant differences based on least significant difference (LSD) (*P* < 0.05).


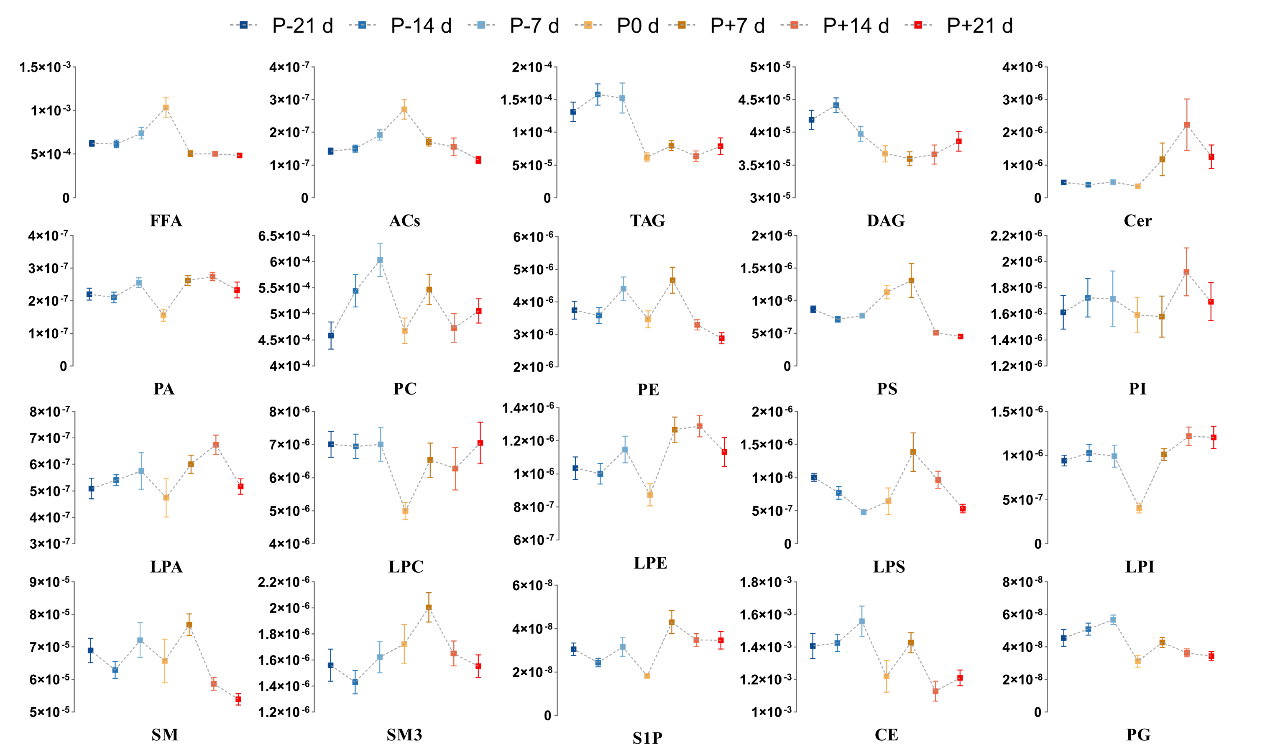
 **Fig. S3** Line graph depicting the peripartal changes for all lipid species levels in dairy goats. **P-21 d**, **P-14 d**, **P-7 d** (d 21,14 and 7 before the due date), **P 0 d** (the day of kidding), and **P+7 d**, **P+14 d**, **P+21 d** (d 7, 14, and 21postpartum). Results are expressed as means ± SEM. **ACs** = acylcarnitine; **CE** = cholesteryl esters; **Cer** = ceramides; **DAG** = diacylglycerols; **FFA** = free fatty acids; **GM3** = monosialogangliosides; **PA** = phosphatidic acids; **PC** = phosphatidylcholines; **LPC** = lyso-PC; **PE** = phosphatidylethanolamines; **PG** = phosphatidylglycerols; **PI** = phosphatidylinositols; **PS** = phosphatidylserines; **S1P** = sphingosine-1-phosphate; **SM** = sphingomyelins; **TAG** = triacylglycerols.


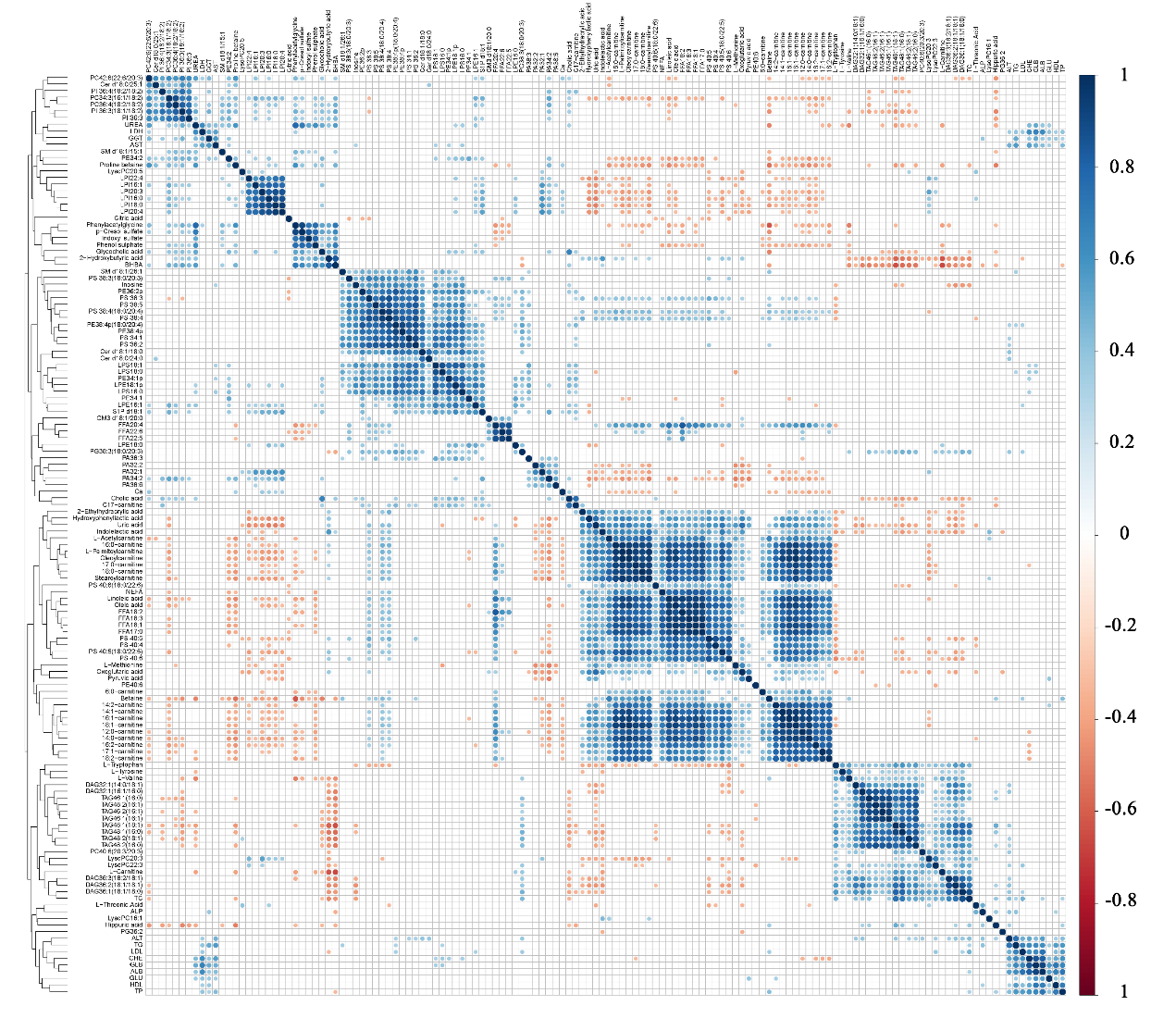
**Fig. S4** Correlations between plasma biochemical indices, differentially altered metabolites, and lipid levels at all time points combined. The left margin shows a dendrogram from hierarchical cluster analysis by which rows and columns are ordered.
